# Dependence of the Staphylococcal Volatilome Composition on Microbial Nutrition

**DOI:** 10.3390/metabo10090347

**Published:** 2020-08-27

**Authors:** Carrie L. Jenkins, Heather D. Bean

**Affiliations:** 1 School of Life Sciences, Arizona State University, Tempe, AZ 85287, USA; Carrie.Jenkins@asu.edu; 2 Center for Fundamental and Applied Microbiomics, The Biodesign Institute, Arizona State University, Tempe, AZ 85287, USA

**Keywords:** *Staphylococcus aureus*, *Staphylococcus epidermidis*, volatile organic compounds, GC×GC-TOFMS, catabolite repression control

## Abstract

In vitro cultivation of staphylococci is fundamental to both clinical and research microbiology, but few studies, to-date, have investigated how the differences in rich media can influence the volatilome of cultivated bacteria. The objective of this study was to determine the influence of rich media composition on the chemical characteristics of the volatilomes of *Staphylococcus aureus* and *Staphylococcus epidermidis*. *S. aureus* (ATCC 12600) and *S. epidermidis* (ATCC 12228) were cultured in triplicate in four rich complex media (brain heart infusion (BHI), lysogeny broth (LB), Mueller Hinton broth (MHB), and tryptic soy broth (TSB)), and the volatile metabolites produced by each culture were analyzed using headspace solid-phase microextraction combined with comprehensive two-dimensional gas chromatography—time-of-flight mass spectrometry (HS-SPME-GC×GC-TOFMS). When comparing the chemical compositions of the staph volatilomes by the presence versus absence of volatiles produced in each medium, we observed few differences. However, when the relative abundances of volatiles were included in the analyses, we observed that culturing staph in media containing free glucose (BHI and TSB) resulted in volatilomes dominated by acids and esters (67%). The low-glucose media (LB and MHB) produced ketones in greatest relative abundances, but the volatilome compositions in these two media were highly dissimilar. We conclude that the staphylococcal volatilome is strongly influenced by the nutritional composition of the growth medium, especially the availability of free glucose, which is much more evident when the relative abundances of the volatiles are analyzed, compared to the presence versus absence.

## 1. Introduction

In vitro cultivation of staphylococci is fundamental to both clinical and research microbiology, and the selection of growth medium substantially influences staph growth rates, genetic integrity, pathogenicity, and metabolic capacity [1,2,3,4,5]. Depending on the species, staphylococci possess remarkable genetic plasticity, regulated by two-component systems that enable rapid responses to fluctuations in environmental conditions [1,4,6]. Additionally, staph is able to survive under host sequestration (also known as nutritional immunity) and microbial competition, both of which influence the availability of essential nutrients and carbon sources [1,4,6,7,8,9,10,11,12,13,14,15]. Like many heterotrophic bacteria, glucose is the preferred carbon and energy source for staphylococci, which employ catabolite repression control to regulate metabolism in glucose-rich environments. Under catabolite repression control in glucose-replete conditions, the tricarboxylic acid cycle is markedly downregulated, forcing catabolism through the Embden–Meyerhof–Parnas pathway (EMP; glycolysis) and the pentose phosphate pathway (PPP) [16,17,18,19]. Once glucose and other hexose sugars are depleted, other nutrient sources, such as peptides, will be imported and catabolized. Alternatively, under starvation conditions, intracellular biomass (amino acids, proteins, RNA) will become substrates of endogenous metabolism [17,20,21].

Four common media used for the enrichment of staph are brain heart infusion (BHI), lysogeny broth (LB), Mueller Hinton broth (MHB), and tryptic soy broth (TSB). All of these are complex media with high contributions of amino acids or peptides to the total organic dry weight, ranging from approximately 66% in LB to 89% in TSB and 93% in BHI and MHB. Thus, these media provide nitrogen-replete conditions for protein and nucleotide synthesis during growth. In contrast, the sugar content of these media substantially differs. TSB and BHI contain 11% and 7% dextrose (α-d-glucose), respectively, by dry organic weight, while MHB provides 7% starch (amylose and amylopectin), which can be hydrolyzed by most staphylococci via α-amylase to generate free sugars for glycolysis [22]. LB contains no added dextrose or starches, and the yeast extract ingredient has been shown to contribute less than 100 µM fermentable sugar equivalents (nucleotides, sugar phosphates, or oligosaccharides), which substantially slow the growth of *Escherichia coli* and *S. aureus* once the sugars are depleted [23,24,25]. A few other notable differences in the media may influence staph metabolism. For instance, MHB has low concentrations of thymine and thymidine, which will require upregulations in thymidine precursor biosynthesis during replication [26,27]. Additionally, the proteins in BHI, LB, and TSB have been enzymatically digested, generating random length oligopeptides, while MHB contains an acid digest of casein, which generates free amino acids that are not as easily imported by staphylococci [28]. Lastly, the low lipid content of TSB, contributing <0.05% dry organic weight, may introduce a differential effect on staph metabolism compared to other media, as both *S. aureus* and *S. epidermidis* must activate de novo fatty acid biosynthetic pathways when exogenous fatty acids are unavailable for assimilation [29,30,31].

We are specifically interested in the role that growth media play on the production of the volatile metabolome (or volatilome) of staph and other microbes. In BHI, LB, MHB, and TSB, proteolysis and/or glycolysis will dominate the production of volatiles. Amino acid catabolism generates sulfur and nitrogen-containing volatiles (e.g., dimethyl disulfide, pyrazines, nitriles, and pyrroles), as well as aromatic compounds (e.g., aromatic alcohols, aldehydes, and ketones) from the catabolism of aromatic amino acids [32,33,34]. Additionally, acids, aldehydes, ketones, and alcohols are formed by amino acid degradation via deamination, followed by decarboxylation and reduction [32,33,34]. Catabolism of glucose to pyruvate and acetyl-CoA leads to the synthesis of short-chain fatty acids and esters, which can be reduced to aldehydes, alcohols, and saturated and unsaturated hydrocarbons [32,33,34]. Extrapolating these observations to predict effects on the volatilome of staph is challenging, though two comparative analyses of bacterial volatiles in rich media have been performed and inform our hypotheses. Dryahina and colleagues [35] analyzed the volatilomes of ten isolates each of *S. aureus*, *Pseudomonas aeruginosa*, *Stenotrophomonas maltophilia*, and *Burkholderia cepacia* complex in BHI, MHB, and nutrient broth (NB), which like LB contains yeast extract and no added sugars or starches. *S. aureus* produced the highest proportion of acids when grown in BHI, the medium with readily accessible sugars, and when grown in the no-sugar medium, NB, it produced the highest proportion of hydrocarbons. Rees and colleagues [36] characterized the volatilomes of *Klebsiella pneumoniae* clinical isolates cultivated in BHI, LB, MHB, and TSB. When comparing the four media by the numbers of compounds produced in each, similar chemical compositions of the *K. pneumoniae* volatilomes were observed. However, when clustering the volatilomes using the relative abundances of the volatiles, they observed significant media-dependent differences in the volatilomes, which clustered by high-glucose media (BHI and TSB) versus low-glucose media (LB and MHB).

The primary goal of this study was to determine how the chemical compositions of the volatilomes of *S. aureus* and *S. epidermidis* are affected by the nutrients of the growth medium. Because glucose is the preferred carbon source of staphylococci, we expected that media with high concentrations of glucose would support the highest overall abundances of volatiles. We also hypothesized that high-glucose media would activate catabolite repression control, and thus a narrower range of metabolic pathways will be engaged, which will result in the production of a lower diversity of volatile chemical classes. Additionally, we expected to observe a higher relative abundance of aldehydes, acids and esters, ketones and alcohols in high-glucose media versus low-glucose media. Conversely, media without glucose will not induce catabolite repression control and will, therefore, have a higher relative abundance of volatiles derived from amino acid catabolism, e.g., nitrogen and sulfur compounds. To test our hypotheses, we cultured *S. aureus* and *S. epidermidis* in BHI, LB, MHB, and TSB and analyzed the volatilomes using headspace solid-phase microextraction combined with comprehensive two-dimensional gas chromatography – time-of-flight mass spectrometry (HS-SPME-GC×GC-TOFMS). We compared the volatilomes by the number and relative abundance of volatiles in each chemical class. Additionally, we compared the analyzed volatilomes to the literature on *S. aureus* and *S. epidermidis* volatiles and added several compounds to the volatilomes of both species by reporting them here for the first time.

## 2. Results and Discussion

### 2.1. Staphylococcal Volatile Metabolites Produced in Rich Media

Aerobic growth of *S. aureus* and *S. epidermidis* in four complex media (BHI, LB, MHB, and TSB) yielded 337 non-redundant peaks (Table S1 in the Supplementary Materials), 85 of which we were able to assign names based on mass spectral and retention index matches to the NIST library and prior published analyses of *Staphylococcus* spp. volatiles (Table 1). We assigned chemical classifications to an additional 61 volatiles that were identified to a Metabolomics Standards Initiative (MSI) level 3 (Table S1). Of the level 2 or level 3 volatiles, 102 were detected in *S. aureus* culture filtrates, 110 were detected in *S. epidermidis* filtrates, and 66 volatiles were produced by both species. We detected seven volatiles in this study, which have been frequently detected in prior analyses of *S. aureus*, *S. epidermidis*, and other staphylococci: 3-methylbutanoic acid, 3-methylbutan-1-ol, 3-methylbutanal, 2-phenylacetaldehyde, butan-2-one, butane-2,3-dione, and undecan-2-one. Sixty-eight compounds from this study were named for the first time as volatile metabolites of either *S. aureus* (n = 9), *S. epidermidis* (n = 37), or both (n = 22), and 12 of them have not been previously described for any bacterium (Table 1).

We identified 36 volatiles that were specific to *S. aureus* and 44 to *S. epidermidis*. Two *S. aureus*-specific volatiles that were detected in the greatest relative abundance in BHI and TSB filtrates were acetic acid and 3-methylbut-3-enyl acetate (isopentenyl alcohol acetate). We posited that the former was resulting from a process called overflow metabolism through the reversible Pta-AckA pathway, in which bacteria will excrete acetate during rapid growth via glycolysis [1,37]. Upon glucose depletion, acetate is assimilated by the same pathway for energy production, and, therefore, the absence of similar amounts of acetate in *S. epidermidis* BHI and TSB cultures may be related to growth kinetics (i.e., sampling at only one time point). 2,4-Dimethyl-1H-pyrrole, a constituent of porphobilinogen, the major precursor of heme and other bacterial siderophores, was detected exclusively in *S. epidermidis* filtrates of BHI and LB in high abundance. We were unable to specify this metabolite as a product of anabolism or catabolism, but one possibility is that the propionic acid portions of porphobilinogen are stripped off as easily available carbon and energy sources by *S. epidermidis*, and the 2,4-dimethyl-1H-pyrrole is discarded as a catabolic byproduct. Additional compounds detected solely in the filtrates of *S. epidermidis* were benzaldehyde (MHB only), 2-phenylacetaldehyde (all media), and (methyldisulfanyl)methane (BHI and LB), though all three compounds have been reported in prior analyses of *S. aureus* volatilomes [38,39,40,41].

### 2.2. Chemical Composition of the S. aureus and S. epidermidis Volatilomes

**Figure 1:**
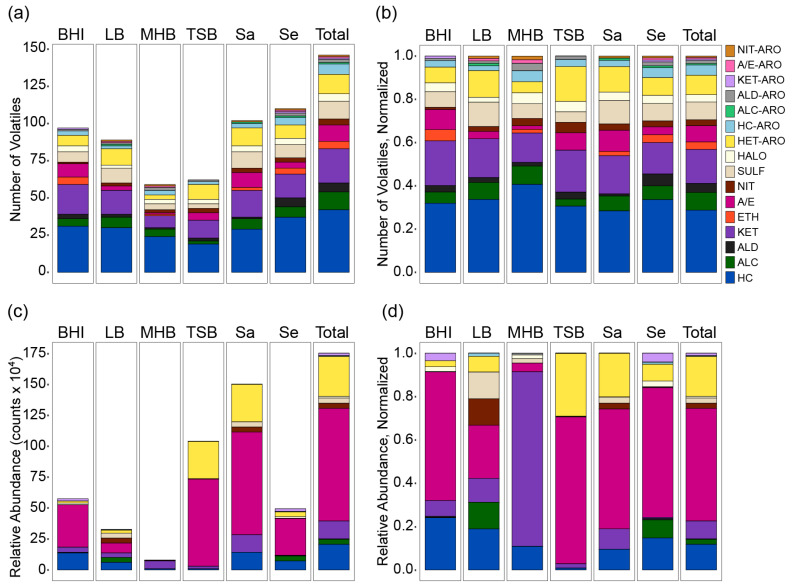
Chemical composition of the total staph volatilome (Total), and the volatilome by species (*S. aureus*, Sa, or *S. epidermidis*, Se) or by growth medium (brain heart infusion (BHI), tryptic soy broth (TSB), Mueller Hinton broth (MHB), lysogeny broth (LB)). The data are presented as the total number of volatiles detected in each chemical class, unnormalized (**a**) and normalized (**b**), and the total relative abundances of volatiles in each chemical class, unnormalized (**c**) and normalized (**d**). Level 4 (unknown) compounds were excluded. Each column represents composite data, with the media volatilomes representing the data for both species, the species volatilomes representing the volatilomes of that species in all four media, and the total representing the volatilomes of both species in all four media. The numerical data are provided in Tables S2–S5. HC, hydrocarbons; ALC, alcohols; ALD, aldehydes; KET, ketones; ETH, ethers; A/E, acids and esters; NIT, nitrogen-containing; SULF, sulfur-containing; HALO, halogen-containing; HET-ARO, heteroaromatics; HC-ARO, aromatic hydrocarbons; ALC-ARO, aromatic alcohols; ALD-ARO, aromatic aldehydes; KET-ARO, aromatic ketones; A/E-ARO, aromatic acids and esters; NIT-ARO, aromatic nitrogen-containing.

We evaluated the composition of the staph volatilome both by the numbers of volatiles in each of the 16 chemical classes (i.e., presence vs. absence) and by the relative abundances of the volatiles in each class using the 146 level 2 and level 3 volatiles. When defining the composition by the number of volatiles, a compound that was detected in any one of the *S. aureus* or *S. epidermidis* cultures in any medium was counted toward the volatilome. When defining the volatilome composition by the relative abundance, the highest peak intensity for each compound across any of the *S. aureus* or *S. epidermidis* cultures was added to the total. By the number of volatiles, the total staph volatilome, which is a combination of *S. aureus* and *S. epidermidis* in all four complex media, was dominated by hydrocarbons (HC, n = 42, 29%), followed by aromatic compounds (aggregated across all aromatic compound categories; ARO, n = 26, 18%), ketones (KET, n = 23, 16%), sulfur compounds (SULF, n = 12, 8%), alcohols (ALC, n = 12, 8%), and acids and esters (A/E, n = 11, 8%) (Figure 1a,b). The composite volatilomes of *S. aureus* and *S. epidermidis* across all four media were quite similar to each other, but the proportions of more oxidized compounds to hydrocarbons was higher in *S. aureus* compared to *S. epidermidis*, with the most pronounced differences observed in the numbers of acids and esters. When characterizing the volatilomes by the relative abundances of the chemical classes, a different profile was revealed in which acids and esters (which are primarily short-chain fatty acids) dominated (Figure 1c,d). Though high-confidence comparisons between class intensities could not be made due to variations in ionization efficiency and a lack of chemical standards, the diminishing contribution of hydrocarbons relative to acids and esters in the abundance-based characterization of the volatilome was notable.

### 2.3. Influence of Medium and Nutrition on the Chemical Composition of the Volatilome

Based on differences in glucose concentration in the four complex media, and its influence on *Staphylococcus* spp. catabolite repression control, we posited that the volatilome of both species would strongly vary by media. Interestingly, whether we characterized the volatilome by numbers of volatiles (Figure 1a,b) or by relative abundance (Figure 1c,d) determined whether we classified this hypothesis as true or false. When viewed by the numbers of volatiles in each chemical class, the staph volatilomes in each medium were relatively uniform (Figure 1a,b). For example, in all four media, hydrocarbons were the most numerous, and hydrocarbons plus ketones made up about 50% of all volatiles detected. However, some notable variations did occur. First, the numbers of volatiles that were produced in each medium did not correlate with the availability of free glucose (Figure 1a). Second, the two high-glucose media had some interesting differences in their volatilome compositions. The TSB volatilome contained the lowest numbers of alcohols and sulfurous volatiles, while BHI produced proportionally high numbers of acids and esters but the fewest aromatics of any kind. LB, a low glucose medium, produced the greatest number of alcohols and sulfurs but the fewest halogens and no detectable ethers. Although MHB cultures had the lowest number of identified analytes, it produced a staph volatilome with the largest percentage of hydrocarbons and the lowest percentage of ketones.

Comparing the staphylococcus volatilomes across the rich media not by numbers of compounds but by the relative abundance of volatiles in each chemical class told a contradictory story (Figure 1c,d). First, the relative abundance of volatiles produced in each medium did trend with the availability of glucose (TSB > BHI > LB > MHB). In addition, while acids and esters comprised 8% of the total number of volatiles detected across all four media (Figure 1b), by relative abundance, they made up approximately half (52%) of all classified volatiles, and heteroaromatics were the second most abundant at 18% (Figure 1d). Hydrocarbons, by contrast, represented only 12% of the volatilome. Additionally, differences between the four media were much more pronounced when the relative abundances of the volatiles were considered, and interesting patterns emerged related to glucose availability and catabolite repression control (Figure 1d). We observed that the two high-glucose media were the primary sources of the acids and esters in the staphylococcal volatilome, with this class representing approximately two-thirds of the volatiles produced in BHI and TSB. By contrast, staph grown in LB and MHB, the two low-glucose media, produced acids and esters at 25% and 4%, respectively. The dependency of acid production on the availability of free glucose was consistent with prior observations of the volatilome of *S. aureus* grown in BHI, MHB, and NB [35]. More strikingly, the overall volatilome compositions of LB and MHB were very different, with the former producing a relatively even distribution of many different chemical classes, and the latter almost exclusively producing ketones, which might be caused by growth defects due to the poor uptake of amino acid monomers in MHB relative to polypeptides in LB by staphylococci [28]. The non-acid/ester portion of the volatilomes produced in high-glucose media were also unique, with TSB dominated by heteroaromatics and BHI by hydrocarbons.

**Figure 2:**
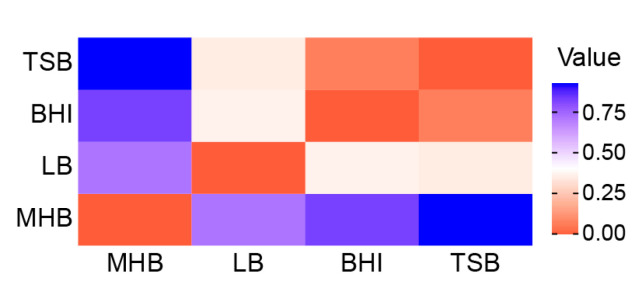
Media-dependent staph volatilome variation represented as a dissimilarity matrix using Morisita–Horn differences calculated using relative abundances of volatile chemical classes.

We quantified the chemical diversities and dissimilarities of the volatilomes in each medium using Shannon diversity and Morisita–Horn methods, respectively, and the relative abundances of volatile chemical classes (Table S5). We observed that the Shannon diversities of the staph volatilomes in BHI and TSB were relatively low (H = 0.84 and 0.73, respectively) compared to LB (H = 1.78), while their dissimilarities to each other were the lowest of any pair-wise comparison (C*_MH_* = 0.071) (Figure 2). Together, these data suggested that adding glucose to the medium would regulate the composition of the staph volatilome by inducing catabolite repression control and enhancing the production of acids and esters. When glucose is unavailable, a wider variety of metabolic pathways will be induced based upon the carbon sources specific to each medium, thus resulting in volatilome compositions that are more unique and possibly more diverse. If true, we predict that the addition of glucose to LB or MHB will enhance the production of acids and esters. We also predict that shorter growth times (e.g., 8 vs. 24 h) will reduce the differences we observed in the non-acid/ester portion of the BHI and TSB volatilomes, which we posit arose after glucose was exhausted from the medium and catabolite repression control was removed. Experiments to test these hypotheses remain for the future.

### 2.4. Species-Specific Responses to Medium Nutrition

**Figure 3:**
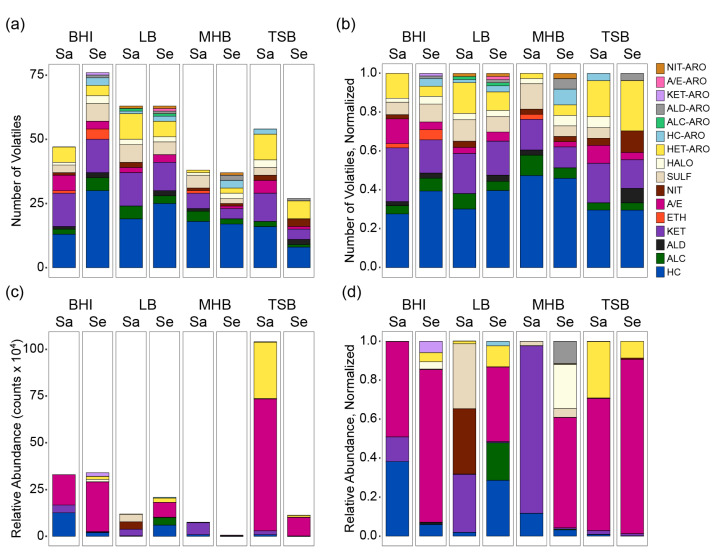
Chemical composition of the staph volatilome as a function of species and growth medium. The data are presented as the total number of volatiles detected in each chemical class, unnormalized (**a**) and normalized (**b**), and the total relative abundances of volatiles in each chemical class, unnormalized (**c**) and normalized (**d**). Level 4 (unknown) compounds were excluded. The numerical data are provided in Tables S2–S5. HC, hydrocarbons; ALC, alcohols; ALD, aldehydes; KET, ketones; ETH, ethers; A/E, acids and esters; NIT, nitrogen-containing; SULF, sulfur-containing; HALO, halogen-containing; HET-ARO, heteroaromatics; HC-ARO, aromatic hydrocarbons; ALC-ARO, aromatic alcohols; ALD-ARO, aromatic aldehydes; KET-ARO, aromatic ketones; A/E-ARO, aromatic acids and esters; NIT-ARO, aromatic nitrogen-containing.

We examined the species-specific responses to the four complex media to obtain a fuller understanding of staphylococcal responses to differences in nutrition (Figure 3). We saw that the number of volatiles produced by each species in each medium did not correspond to free glucose availability (Figure 3a), demonstrating that our prior observation of the staph volatiles was not a function of data aggregation (Figure 1a). We also observed that the volatilomes of both species were dominated by high numbers of hydrocarbons and ketones in all media, and in each medium, the ratio of hydrocarbons to ketones was larger in *S. epidermidis* than in *S. aureus*. When analyzing the volatilomes by the relative abundances of each chemical class (Figure 3c), we found that the relative abundance of *S. aureus* volatiles correlated to free glucose availability (TSB > BHI > LB > MHB), but the *S. epidermidis* volatilome did not (BHI > LB > TSB > MHB). We also observed that *S. aureus* seemed to be more strongly influenced by the presence or absence of free glucose, with half or more of the volatilome represented by acids and esters in the high glucose media and less than one percent in the low glucose media (Figure 3d). *S. epidermidis*, by contrast, produced abundant acids and esters in all four media, but at 78–89% of the total abundance in high-glucose media, and 38–57% in the low glucose media. Because we did not have time course sampling of the volatilomes, we could not determine if these differences were a function of greater relative production of acids and esters by *S. epidermidis* in all four media or reduced assimilation as an alternate carbon source in nutrient-depleted media. However, these snapshots supported the assertion by Filipiak and colleagues [64] who surmise that attempts to standardize optimal cultivation conditions by the selection of a single medium for all microorganisms—even two species of the same genus, as shown here—will insufficiently reveal the full spectrum of volatile metabolic compounds.

## 3. Materials and Methods

### 3.1. Bacterial Strains, Culture Conditions, and Metabolomics Analysis Sample Preparation

The volatilomes of *S. aureus* (ATCC 12600) and *S. epidermidis* (ATCC 12228) were analyzed after 24 h aerobic incubation at 37 °C in the following four filter-sterilized complex media: brain heart infusion broth (BHI) (Becton, Dickenson and Co., Franklin Lakes, NJ, USA) [77], lysogeny broth Lennox (LB) (Becton, Dickenson and Co., Franklin Lakes, NJ, USA), Mueller Hinton broth (MHB) —not cation-adjusted (Becton, Dickenson and Co., Franklin Lakes, NJ, USA) [77], and tryptic soy broth (TSB) (Becton, Dickenson and Co., Franklin Lakes, NJ, USA) [77]. The manufacturers’ part numbers and compositions of the media are in Table S6. The bacteria were pre-cultured on LB agar for 24 h at 37 °C, and single colonies were inoculated into 5 mL of BHI, LB, MHB, or TSB media in 50 mL conical tubes, which were incubated overnight in an orbital shaker at 37 °C and 200 rpm. The overnight cultures were subcultured with a 1000-fold dilution into 25 mL media in 250 mL Erlenmeyer flasks with foam stoppers and incubated for 24 h in an orbital shaker at 37 °C and 200 rpm. Bacterial cultures were prepared in biological triplicates, and media-only controls were prepared in six replicates for BHI, LB, and MHB, and in triplicate for TSB, following the same procedures as the bacterial cultures. After culturing, the samples and blanks were chilled on ice, then centrifuged to pellet the cells. Ten milliliters of the supernatants were filter-sterilized at 4 °C through Macrosep Advance centrifugal filter devices (Pall, 0.20 μm Supor membrane, New York, NY, USA) that were pre-rinsed using 10 mL filter-sterilized media. Two milliliters of each filtrate were transferred to heat-treated (24 h at 100°C) 10 mL GC headspace vials with polytetrafluoroethylene/silicone septum screw caps. The samples were stored for approximately two weeks at −20 °C prior to volatile metabolomics analysis.

### 3.2. Analysis by HS-SPME-GC×GC-TOFMS

Headspace solid-phase microextraction (HS-SPME), chromatographic and mass spectrometric analyses, and data processing and alignment were carried out on a Leco Pegasus 4D GC×GC-TOFMS (St. Joseph, MI, USA) outfitted with a Gerstel MultiPurpose Sampler autosampler (Mülheim, Germany), as previously published [78], using parameters in Table S7. Samples and media blanks were analyzed in a randomized order. The mass spectrometer was calibrated daily with perfluorotributylamine, and the column performance was checked at three points during the analysis using a quality control sample (a 24 h culture of *Pseudomonas aeruginosa* PA14 in LB, sampled using the parameters described in Table S7) and at the beginning, mid-point, and end of the run using alkane standards (C_6_-C_15_; Sigma-Aldrich). Headspace volatiles of a pure alkane mixture were sampled using a 2 cm divinylbenzene/carboxen/polydimethylsiloxane (DVB/CAR/PDMS) SPME fiber (Supelco) for 10 min at 50 °C and desorbed for 10 min at 250 °C without a split. All other GC×GC-TOFMS parameters for the alkane mix were as described in Table S7. The alkane retention times were utilized to calculate retention indices (RIs); RIs greater than 1500 or less than 600 were extrapolated.

The datasets generated during the current study are available as Study ST001426 in the Metabolomics Workbench repository [79], http://dx.doi.org/10.21228/M88Q44.

### 3.3. Statistical Analyses

Peaks were removed that eluted prior to acetone in the first dimension, at 358 s. Probabilistic quotient normalization (PQN), followed by log_10_ transformation, was employed to minimize differences in peak abundance resulting from cell culture density variations [80]. As previously described [78], peaks were excluded upon failure to meet all of the following criteria in at least one sample: (1) present in all biological replicates, (2) two-fold or greater mean peak area compared to the mean peak area in the corresponding media control sample, and (3) significantly greater in abundance in the biological sample versus media control as evaluated by a one-tailed *t*-test (*p* < 0.05). Statistical analyses were performed using RStudio for desktop version 1.3.959 (RStudio: Integrated Development for R. RStudio, PBC). Shannon diversity indices and volatilome dissimilarities were calculated for the relative abundance volatilome data using the Vegan package (v. 2.5-6) with the functions—*diversity* (method: Shannon) and *vegdist* (method: Morisita-Horn).

### 3.4. Compound Identification

Peaks were assigned putative identifications based on mass spectral and retention time data, and the confidence of the identification was indicated using the four levels described by the Chemical Analysis Working Group Metabolomics Standards Initiative (MSI) [81,82]. Level 2 was the highest in this study, indicating a forward and/or reverse NIST MS library match ≥ 800 (out of 1000), and RIs were consistent with published values. The RIs reported here fell between the literature values for polar and nonpolar column sets, as a result of the mid-polarity of the cyanopropyl stationary phase. We considered the measured (experimental) RIs to be consistent with the literature values if they fell within 0–35% of the range between polar and non-polar values (see [65] for calculation).

## 4. Conclusions

The staphylococcal volatilome was strongly influenced by the nutritional composition of the growth medium, which was much more evident when the relative abundances of the volatiles were analyzed, compared to the presence versus absence. In the glucose-free media of LB and MHB, we observed substantial media-dependent differences in the volatilome chemical composition, while in BHI and TSB media containing free glucose, half or more of the volatilome was comprised of acids and esters, and the remaining portion was of unique chemical composition by the medium. We attributed these observations to *S. aureus* and *S. epidermidis* catabolite repression control, which would preferentially catabolize glucose when available and would catabolize a wider variety of substrates (amino acids, organic acids, nucleic acid and nucleotides, alcohols, and heteroaromatics) when glucose is depleted. In the low glucose media LB and MHB, ketones became the most abundant chemical class, suggesting that ketogenic amino acid metabolism dominated under these conditions. Our observations on the relative abundances of acids/esters and ketones versus other chemical classes in any given medium would be very specific to the use of SPME headspace analysis, gas chromatography separations on underivatized analytes, and electron impact ionization. However, these analyses provided preliminary data on how the microbial volatilome could be influenced by microbial nutrition and suggested that the concentration of free glucose in in vitro media should be matched to each infection site to improve the translatability of the volatilomes. Our data also underscored the need to consider volatile abundances in addition to presence and absence when studying the effect of nutrition on volatile metabolites. Future analyses will include experiments to test our hypotheses about the role of catabolite repression control and glucose availability on the staphylococcal volatilome, with an eye toward developing in vitro culture conditions that more closely replicate in vivo volatilomes. We will also explore if the species-specific volatilome responses to free glucose can be correlated to virulence, potentially enabling the detection of pathogenic strains of *S. epidermidis* and other coagulase-negative staphylococci that are typically regarded as commensals.

## Figures and Tables

**Table 1:** Volatiles detected in the headspace of *S. aureus* or *S. epidermidis* cultured in brain heart infusion (BHI), lysogeny broth (LB), Mueller Hinton broth (MHB), or tryptic soy broth (TSB). Peak relative abundances are reported as the Log_10_ fold change of the mean peak intensity in the bacterial culture versus the mean intensity in the corresponding medium control. Italicized volatiles have not been previously reported for *S. aureus* or *S. epidermidis*, and bolded italicized volatiles have not been previously reported for bacteria. Additional data for these volatiles are available in Table S1.

Log_10_ Peak Relative Abundances								Compound Name	CAS #	References
***S. aureus***				***S. epidermidis***						
**BHI**	**LB**	**MHB**	**TSB**	**BHI**	**LB**	**MHB**	**TSB**			
**ACIDS and ESTERS**										
				4.7	4.9	3.5		2-methylbutanoic acid	116-53-0	[41,42,43,44,45]
			4.1	4.2				*2-methylbutyl acetate*	624-41-9	[46,47]
4.3			4.6					3-methylbut-3-enyl acetate	5205-07-2	[38,48]
	2.3		5.8	5.3	2.8		5.0	3-methylbutanoic acid	503-74-2	[36,38,39,40,41,42,43,44,45,46,47,49,50,51,52]
4.2			4.3					acetic acid	64-19-7	[35,38,39,40,41,42,45,46,47,48,53,54,55,56,57,58]
5.1								butyl 2-methylbutanoate	15706-73-7	[51,59]
0.6								*butyl 2-methylpropanoate*	97-87-0	[59]
0.6								butyl acetate	123-86-4	[38,46,47,48,51,59,60]
0.8								***butyl propanoate***	590-01-2	
	0.6				0.6			*dec-5-en-1-ol acetic acid*		[60]
**AROMATIC ACIDS and ESTERS**										
					0.8			methyl benzoate	93-58-3	[38,58]
**ALCOHOLS**										
			1.8					*2-butyloctan-1-ol*	3913-02-8	
					4.6			2-methylbutan-1-ol	137-32-6	[40,43,61,62]
				0.4				2-methylbutan-2-ol	75-85-4	[60,63]
				0.5				2-methylpropan-2-ol	75-65-0	[36,52,64]
				0.4				3-methylbut-3-en-1-ol	763-32-6	[36,38,65]
0.9	0.8	0.6	1.1	2.8	2.5	1.3	1.5	3-methylbutan-1-ol	123-51-3	[36,38,39,40,42,44,46,47,48,51,52,56,60,61,62,64,66,67,68,69,70]
	0.6	0.5						6-methylheptan-1-ol	1653-40-3	[38]
		0.4						octan-1-ol	111-87-5	[38,43,50,55,61]
**AROMATIC ALCOHOLS**										
	0.4				0.4			***2-phenylpropan-2-ol***	617-94-7	
**ALDEHYDES**										
							1.0	2-methylidenebutanal	922-63-4	[47]
					0.3			2-methylpropanal	78-84-2	[36,38,39,46,47,48,57]
				0.3				3-methylbutanal	590-86-3	[40,42,45,46,47,48,50,52,55,62,67,68]
				3.5				oct-2-enal	2363-89-5	[64]
**AROMATIC ALDEHYDES**										
						2.8		benzaldehyde	100-52-7	[38,39,40,41,42,46,47,54,62,71]
				1.1	0.7	1.1	0.5	2-phenylacetaldehyde	122-78-1	[42,52,71]
**ETHERS**										
				0.4				*2-butoxyethanol*	111-76-2	
				0.4				***2-methyl-2-propan-2-yloxypropane***	17348-59-3	
**HYDROCARBONS**										
				0.5				1-methyl-4-prop-1-en-2-ylcyclohexene	138-86-3	[38]
1.2					1.8	0.9		***2,4,6,8-tetramethylundec-1-ene***	59920-26-2	
	0.7	1.0		0.7	1.0	0.7	0.4	*3,3-dimethyloctane*	4110-44-5	[60]
1.0	1.4	1.6	1.5	1.2	1.7	1.0	0.8	***3,7-dimethyloct-1-ene***	4984-01-4	
				0.4				4-methylheptane	589-53-7	[36,48]
	3.3		3.9					***7-methyl-3-methylideneocta-1,6-diene***	123-35-3	
1.4	1.2	1.5		1.8	1.8	1.0	0.6	*decane*	124-18-5	[48,65]
	1.3							***dodecane***	112-40-3	
		0.7		0.9				***hexan-2-ylcyclopropane***	6976-28-9	
1.0	1.3	2.3	1.2	1.2	1.5	1.4	0.4	***tridec-3-ene***		
		0.9		0.3				***undec-3-ene***	60669-40-1	
				0.7	0.6			undecane	1120-21-4	[36,48,66,72]
**AROMATIC HYDROCARBONS**										
					3.7			***1,1,5,6-tetramethyl-2H-naphthalene***	220766-68-7	
				0.4		0.3		*1,2-xylene*	95-47-6	
	0.3				0.4			***1,3-ditert-butylbenzene***	1014-60-4	
				0.4		0.3		*1,4-xylene*	106-42-3	
			0.3					*1-ethyl-2-methylbenzene*	611-14-3	[43]
				0.4		0.3		*ethylbenzene*	100-41-4	[36,48,73]
**KETONES**										
1.1	0.7		0.9	1.1				*3-methylbutan-2-one*	563-80-4	[36,60,63,64,65]
0.6								*4,6-dimethylheptan-2-one*	19549-80-5	[36]
1.7	2.7	1.6	1.6	1.7	3.0			4-methylheptan-2-one	6137-06-0	[36,38,39]
	0.9		0.8	0.8	0.6			4-methylpentan-2-one	108-10-1	[38,46,65]
	0.4							6-methylhept-5-en-2-one	110-93-0	[38,48,71]
2.0	1.9			0.9	1.1			*6-methylheptan-2-one*	928-68-7	[36,38]
				0.3	0.3			but-3-en-2-one	78-94-4	[46,48,65,74]
0.4								butan-2-one	78-93-3	[35,36,38,42,46,55,56,62,63,64,65,66,69,72,75]
0.5			1.6		0.5			butane-2,3-dione	431-03-8	[36,46,47,48,53,55,60,62,64,74,75,76]
				0.4				cyclohexanone	108-94-1	[38,43,48]
2.3	2.9			1.0	1.6			*decan-2-one*	693-54-9	[38,65]
				1.2		0.7		dodecan-2-one	6175-49-1	[65]
	0.4		1.9	1.1	1.3		0.7	heptane-2,3-dione	96-04-8	[38,64,68]
1.6	1.4	0.7	1.6	0.6	1.0			nonan-2-one	821-55-6	[35,36,38,42,43,44,46,48,49,50,55,60,61,64,65,68,73]
2.9	2.8	2.1	2.7	1.6	1.6	0.8	0.5	octan-2-one	111-13-7	[35,38,67,68,73]
	1.2		3.6	0.9	1.3	1.3	3.1	octan-3-one	106-68-3	[38,46,48,65]
0.7			0.6					pentan-2-one	107-87-9	[35,38,39,42,45,46,47,48,52,53,56,62,64,65]
					0.5			propan-2-one	67-64-1	[35,38,48,55,56,57,60,65,66,72,74]
2.7	3.6	2.8				0.5		tetradecan-2-one	2345-27-9	[38]
2.4	4.5	2.0	2.1					*tridecan-2-one*	593-08-8	[38,61]
	2.8	4.8	4.2					undecan-2-one	112-12-9	[38,39,42,49,50,61,64,65,68]
**AROMATIC KETONES**										
				4.3				1-phenylpropan-2-one	103-79-7	[36,38,73]
**NITROGENS**										
			2.5			0.7	0.5	***3-methylbutanenitrile***	625-28-5	
**AROMATIC NITROGENS**										
	0.4				0.4	0.5		benzonitrile	100-47-0	[36,38,64,68,73]
**HETEROAROMATICS**										
0.7	1.0		2.5	0.5	1.2		0.8	*2,3,5-trimethylpyrazine*	14667-55-1	[41,44,62,68]
0.4								*2,3-dimethylpyrazine*	5910-89-4	[41,73]
				4.2	4.0			***2,4-dimethyl-1H-pyrrole***	625-82-1	
1.4	2.0		2.3	0.5	1.0	1.0	1.1	2,5-dimethylpyrazine	123-32-0	[43,44,53,62,71]
			3.3				2.8	*2-ethyl-6-methylpyrazine*	13925-03-6	[66]
	1.5		1.2		1.2	0.7	1.3	*2-methyl-5-propylpyrazine*	29461-03-8	
0.5	0.7	0.9	1.5	0.3	0.4		0.7	*2-methylpyrazine*	109-08-0	[36,41,44,62,64,68]
	0.3		4.3				3.0	*3,5-dimethyl-2-(3-methylbutyl)pyrazine*	111150-30-2	[73]
0.5	1.7		3.2					*3-butyl-2,5-dimethylpyrazine*	40790-29-2	
	2.7		3.8		4.1			3-ethyl-2,5-dimethylpyrazine	13360-65-1	
**SULFURS**										
			0.8	1.0	1.1			2-methylundecane-2-thiol	10059-13-9	[38,71]
0.4	0.7			0.3				***isothiocyanatocyclohexane***	1122-82-3	
				0.4	0.4			(methyldisulfanyl)methane	624-92-0	[35,36,38,41,46,47,48,55,56,58,61,62,65,66,67,68,74]
0.5		0.4		0.5	0.6	0.4		(methyltrisulfanyl)methane	3658-80-8	[35,38,46,48,55,61,65,66,67,68]

## Supplementary Materials

The following are available online at https://www.mdpi.com/2218-1989/10/9/347/s1: Table S1: Volatile compounds detected in the headspace of in vitro cultures of *S. aureus* or *S. epidermidis* cultured in BHI, LB, MHB, or TSB, Table S2: Number of volatiles produced in 16 chemical classes by culturing *S. aureus* or *S. epidermidis* in BHI, LB, MHB, or TSB, Table S3: Normalized number of volatiles produced in 16 chemical classes by culturing *S. aureus* or *S. epidermidis* in BHI, LB, MHB, or TSB, Table S4: Relative abundances of volatiles produced in 16 chemical classes by culturing *S. aureus* or *S. epidermidis* in BHI, LB, MHB, or TSB, Table S5: Normalized relative abundances of volatiles produced in 16 chemical classes by culturing *S. aureus* or *S. epidermidis* in BHI, LB, MHB, or TSB, Table S6: Media composition and characteristics, Table S7: Parameters for HS-SPME volatiles sampling, GC×GC-TOFMS analysis, and data processing and alignment.
