# Supplementary material for: Dependence of the Staphylococcal Volatilome Composition on Microbial Nutrition
Source: Metabolites. 2020 Aug 27;10(9):347. doi: 10.3390/metabo10090347 (PMC7569959; doi:10.3390/metabo10090347)
Supplement: Supplementary file 1 [file metabolites-10-00347-s001.zip › Jenkins_SupplementaryInformation_Final.pdf]

**Supplementary Information**

for

# Dependence of the Staphylococcal Volatilome Composition on Microbial Nutrition

Carrie L. Jenkins and Heather D. Bean

**Table S1** See **Supplementary File**. Volatile compounds detected in the headspace of *in vitro* cultures of *S. aureus* (Sa) or *S. epidermidis* (Se) cultured in brain heart infusion (BHI), lysogeny broth Lennox (LB), Mueller-Hinton broth (MHB), or tryptic soy broth (TSB).

**Table S2** Number of volatiles produced in 16 chemical classes by culturing *S. aureus* (Sa) or *S. epidermidis* (Se) in brain heart infusion (BHI), lysogeny broth (LB), Mueller-Hinton broth (MHB), or tryptic soy broth (TSB). A list of abbreviations for the chemical classes is provided in Table S1.

| | NIT-ARO | A/E-ARO | KET-ARO | ALD-ARO | ALC-ARO | HC-ARO | HET-ARO | HALO | SULF | NIT | A/E | ETH | KET | ALD | ALC | HC | Total |
|---|---|---|---|---|---|---|---|---|---|---|---|---|---|---|---|---|---|
| **BHI, Sa** | 0 | 0 | 0 | 0 | 0 | 0 | 6 | 1 | 3 | 1 | 6 | 1 | 13 | 1 | 2 | 13 | 47 |
| **BHI, Se** | 0 | 0 | 1 | 1 | 0 | 3 | 4 | 3 | 7 | 0 | 3 | 4 | 13 | 2 | 5 | 30 | 76 |
| **LB, Sa** | 1 | 0 | 0 | 0 | 1 | 1 | 10 | 2 | 7 | 2 | 2 | 0 | 13 | 0 | 5 | 19 | 63 |
| **LB, Se** | 1 | 1 | 0 | 1 | 1 | 2 | 6 | 2 | 5 | 0 | 3 | 0 | 11 | 2 | 3 | 25 | 63 |
| **MHB, Sa** | 0 | 0 | 0 | 0 | 0 | 0 | 1 | 1 | 5 | 1 | 0 | 1 | 6 | 1 | 4 | 18 | 38 |
| **MHB, Se** | 1 | 0 | 0 | 2 | 0 | 3 | 2 | 2 | 2 | 1 | 1 | 0 | 4 | 0 | 2 | 17 | 37 |
| **TSB, Sa** | 0 | 0 | 0 | 0 | 0 | 2 | 10 | 3 | 3 | 2 | 5 | 0 | 11 | 0 | 2 | 16 | 54 |
| **TSB, Se** | 0 | 0 | 0 | 1 | 0 | 0 | 7 | 0 | 0 | 3 | 1 | 0 | 4 | 2 | 1 | 8 | 27 |
| **BHI** | 0 | 0 | 1 | 1 | 0 | 3 | 7 | 4 | 7 | 1 | 9 | 5 | 20 | 3 | 5 | 31 | 97 |
| **LB** | 1 | 1 | 0 | 1 | 1 | 2 | 11 | 2 | 10 | 2 | 3 | 0 | 16 | 2 | 7 | 30 | 89 |
| **MHB** | 1 | 1 | 0 | 2 | 0 | 3 | 3 | 3 | 4 | 2 | 1 | 1 | 8 | 1 | 5 | 24 | 59 |
| **TSB** | 0 | 0 | 0 | 1 | 0 | 2 | 10 | 3 | 3 | 3 | 5 | 0 | 12 | 2 | 2 | 19 | 62 |
| **Sa** | 1 | 0 | 0 | 0 | 1 | 3 | 12 | 4 | 11 | 3 | 10 | 2 | 18 | 1 | 7 | 29 | 102 |
| **Se** | 1 | 1 | 1 | 2 | 1 | 5 | 9 | 4 | 9 | 3 | 4 | 4 | 16 | 6 | 7 | 37 | 110 |
| **Total** | 1 | 1 | 1 | 2 | 1 | 7 | 13 | 5 | 12 | 4 | 11 | 5 | 23 | 6 | 12 | 42 | 146 |

**Table S3** Number of volatiles produced in 16 chemical classes by culturing *S. aureus* (Sa) or *S. epidermidis* (Se) in brain heart infusion (BHI), lysogeny broth (LB), Mueller-Hinton broth (MHB), or tryptic soy broth (TSB), normalized to the total number in each row in Table S2. A list of abbreviations for the chemical classes is provided in Table S1.

| | NIT-ARO | A/E-ARO | KET-ARO | ALD-ARO | ALC-ARO | HC-ARO | HET-ARO | HALO | SULF | NIT | A/E | ETH | KET | ALD | ALC | HC |
|---|---|---|---|---|---|---|---|---|---|---|---|---|---|---|---|---|
| **BHI, Sa** | 0.00 | 0.00 | 0.00 | 0.00 | 0.00 | 0.00 | 0.13 | 0.02 | 0.06 | 0.02 | 0.13 | 0.02 | 0.28 | 0.02 | 0.04 | 0.28 |
| **BHI, Se** | 0.00 | 0.00 | 0.01 | 0.01 | 0.00 | 0.04 | 0.05 | 0.04 | 0.09 | 0.00 | 0.04 | 0.05 | 0.17 | 0.03 | 0.07 | 0.39 |
| **LB, Sa** | 0.02 | 0.00 | 0.00 | 0.00 | 0.02 | 0.02 | 0.16 | 0.03 | 0.11 | 0.03 | 0.03 | 0.00 | 0.21 | 0.00 | 0.08 | 0.30 |
| **LB, Se** | 0.02 | 0.02 | 0.00 | 0.02 | 0.02 | 0.03 | 0.10 | 0.03 | 0.08 | 0.00 | 0.05 | 0.00 | 0.17 | 0.03 | 0.05 | 0.40 |
| **MHB, Sa** | 0.00 | 0.00 | 0.00 | 0.00 | 0.00 | 0.00 | 0.03 | 0.03 | 0.13 | 0.03 | 0.00 | 0.03 | 0.16 | 0.03 | 0.11 | 0.47 |
| **MHB, Se** | 0.03 | 0.00 | 0.00 | 0.05 | 0.00 | 0.08 | 0.05 | 0.05 | 0.05 | 0.03 | 0.03 | 0.00 | 0.11 | 0.00 | 0.05 | 0.46 |
| **TSB, Sa** | 0.00 | 0.00 | 0.00 | 0.00 | 0.00 | 0.04 | 0.19 | 0.06 | 0.06 | 0.04 | 0.09 | 0.00 | 0.20 | 0.00 | 0.04 | 0.30 |
| **TSB, Se** | 0.00 | 0.00 | 0.00 | 0.04 | 0.00 | 0.00 | 0.26 | 0.00 | 0.00 | 0.11 | 0.04 | 0.00 | 0.15 | 0.07 | 0.04 | 0.30 |
| **BHI** | 0.00 | 0.00 | 0.01 | 0.01 | 0.00 | 0.03 | 0.07 | 0.04 | 0.07 | 0.01 | 0.09 | 0.05 | 0.21 | 0.03 | 0.05 | 0.32 |
| **LB** | 0.01 | 0.01 | 0.00 | 0.01 | 0.01 | 0.02 | 0.12 | 0.02 | 0.11 | 0.02 | 0.03 | 0.00 | 0.18 | 0.02 | 0.08 | 0.34 |
| **MHB** | 0.02 | 0.02 | 0.00 | 0.03 | 0.00 | 0.05 | 0.05 | 0.05 | 0.07 | 0.03 | 0.02 | 0.02 | 0.14 | 0.02 | 0.08 | 0.41 |
| **TSB** | 0.00 | 0.00 | 0.00 | 0.02 | 0.00 | 0.03 | 0.16 | 0.05 | 0.05 | 0.05 | 0.08 | 0.00 | 0.19 | 0.03 | 0.03 | 0.31 |
| **Sa** | 0.01 | 0.00 | 0.00 | 0.00 | 0.01 | 0.03 | 0.12 | 0.04 | 0.11 | 0.03 | 0.10 | 0.02 | 0.18 | 0.01 | 0.07 | 0.28 |
| **Se** | 0.01 | 0.01 | 0.01 | 0.02 | 0.01 | 0.05 | 0.08 | 0.04 | 0.08 | 0.03 | 0.04 | 0.04 | 0.15 | 0.05 | 0.06 | 0.34 |
| **Total** | 0.01 | 0.01 | 0.01 | 0.01 | 0.01 | 0.05 | 0.09 | 0.03 | 0.08 | 0.03 | 0.08 | 0.03 | 0.16 | 0.04 | 0.08 | 0.29 |

**Table S4** Relative abundances of volatiles produced in 16 chemical classes by culturing *S. aureus* (Sa) or *S. epidermidis* (Se) in brain heart infusion (BHI), lysogeny broth (LB), Mueller-Hinton broth (MHB), or tryptic soy broth (TSB). A list of abbreviations for the chemical classes is provided in Table S1.

| | NIT-ARO | A/E-ARO | KET-ARO | ALD-ARO | ALC-ARO | HC-ARO | HET-ARO | HALO | SULF | NIT | A/E | ETH | KET | ALD | ALC | HC | Total |
|---|---|---|---|---|---|---|---|---|---|---|---|---|---|---|---|---|---|
| **BHI, Sa** | 0 | 0 | 0 | 0 | 0 | 0 | 40 | 11 | 284 | 68 | 161709 | 4 | 41786 | 3 | 28 | 126053 | 329986 |
| **BHI, Se** | 0 | 0 | 19953 | 11 | 0 | 8 | 15857 | 12983 | 778 | 0 | 266498 | 10 | 179 | 3164 | 730 | 20131 | 340302 |
| **LB, Sa** | 2 | 0 | 0 | 0 | 3 | 2 | 1505 | 39 | 39936 | 39816 | 204 | 0 | 35495 | 0 | 44 | 2259 | 119305 |
| **LB, Se** | 2 | 7 | 0 | 5 | 2 | 4838 | 22635 | 96 | 145 | 0 | 80099 | 0 | 1224 | 5 | 40173 | 59903 | 209134 |
| **MHB, Sa** | 0 | 0 | 0 | 0 | 0 | 0 | 7 | 45 | 1656 | 5 | 0 | 2 | 63912 | 3 | 16 | 8671 | 74317 |
| **MHB, Se** | 3 | 0 | 0 | 642 | 0 | 7 | 14 | 1266 | 255 | 5 | 3162 | 0 | 34 | 0 | 25 | 176 | 5589 |
| **TSB, Sa** | 0 | 0 | 0 | 0 | 0 | 1587 | 301531 | 2041 | 215 | 416 | 704277 | 0 | 20646 | 0 | 81 | 8751 | 1039545 |
| **TSB, Se** | 0 | 0 | 0 | 3 | 0 | 0 | 9616 | 0 | 0 | 809 | 100000 | 0 | 1276 | 44 | 32 | 26 | 111806 |
| **BHI** | 0 | 0 | 19953 | 11 | 0 | 8 | 15889 | 12995 | 659 | 68 | 342286 | 15 | 41844 | 3168 | 730 | 138096 | 575722 |
| **LB** | 2 | 0 | 0 | 5 | 3 | 4838 | 23600 | 96 | 40380 | 39816 | 80099 | 0 | 36093 | 5 | 40210 | 61935 | 327082 |
| **MHB** | 3 | 7 | 0 | 642 | 0 | 7 | 22 | 1311 | 1638 | 11 | 3162 | 2 | 63936 | 3 | 38 | 8654 | 79436 |
| **TSB** | 0 | 0 | 0 | 3 | 0 | 1587 | 301536 | 2041 | 215 | 1211 | 704277 | 0 | 20656 | 44 | 99 | 8760 | 1040429 |
| **Sa** | 2 | 0 | 0 | 0 | 3 | 1589 | 301551 | 2110 | 41960 | 40227 | 830188 | 6 | 142016 | 3 | 121 | 143233 | 1503009 |
| **Se** | 3 | 7 | 19953 | 642 | 2 | 4845 | 38064 | 13067 | 1132 | 811 | 295816 | 10 | 2514 | 3272 | 40581 | 72876 | 493595 |
| **Total** | 3 | 7 | 19953 | 642 | 3 | 6433 | 323680 | 13111 | 42190 | 41022 | 912931 | 15 | 142624 | 3272 | 40668 | 207642 | 1754196 |

**Table S5** Relative abundances of volatiles produced in 16 chemical classes by culturing *S. aureus* (Sa) or *S. epidermidis* (Se) in brain heart infusion (BHI), lysogeny broth (LB), Mueller-Hinton broth (MHB), or tryptic soy broth (TSB), normalized to the total abundance in each row in Table S4. A list of abbreviations for the chemical classes is provided in Table S1.

| | NIT-ARO | A/E-ARO | KET-ARO | ALD-ARO | ALC-ARO | HC-ARO | HET-ARO | HALO | SULF | NIT | A/E | ETH | KET | ALD | ALC | HC |
|---|---|---|---|---|---|---|---|---|---|---|---|---|---|---|---|---|
| **BHI, Sa** | 0.00 | 0.00 | 0.00 | 0.00 | 0.00 | 0.00 | 0.00 | 0.00 | 0.00 | 0.00 | 0.49 | 0.00 | 0.13 | 0.00 | 0.00 | 0.38 |
| **BHI, Se** | 0.00 | 0.00 | 0.06 | 0.00 | 0.00 | 0.00 | 0.05 | 0.04 | 0.00 | 0.00 | 0.78 | 0.00 | 0.00 | 0.01 | 0.00 | 0.06 |
| **LB, Sa** | 0.00 | 0.00 | 0.00 | 0.00 | 0.00 | 0.00 | 0.01 | 0.00 | 0.33 | 0.33 | 0.00 | 0.00 | 0.30 | 0.00 | 0.00 | 0.02 |
| **LB, Se** | 0.00 | 0.00 | 0.00 | 0.00 | 0.00 | 0.02 | 0.11 | 0.00 | 0.00 | 0.00 | 0.38 | 0.00 | 0.01 | 0.00 | 0.19 | 0.29 |
| **MHB, Sa** | 0.00 | 0.00 | 0.00 | 0.00 | 0.00 | 0.00 | 0.00 | 0.00 | 0.02 | 0.00 | 0.00 | 0.00 | 0.86 | 0.00 | 0.00 | 0.12 |
| **MHB, Se** | 0.00 | 0.00 | 0.00 | 0.11 | 0.00 | 0.00 | 0.00 | 0.23 | 0.05 | 0.00 | 0.57 | 0.00 | 0.01 | 0.00 | 0.00 | 0.03 |
| **TSB, Sa** | 0.00 | 0.00 | 0.00 | 0.00 | 0.00 | 0.00 | 0.29 | 0.00 | 0.00 | 0.00 | 0.68 | 0.00 | 0.02 | 0.00 | 0.00 | 0.01 |
| **TSB, Se** | 0.00 | 0.00 | 0.00 | 0.00 | 0.00 | 0.00 | 0.09 | 0.00 | 0.00 | 0.01 | 0.89 | 0.00 | 0.01 | 0.00 | 0.00 | 0.00 |
| **BHI** | 0.00 | 0.00 | 0.03 | 0.00 | 0.00 | 0.00 | 0.03 | 0.02 | 0.00 | 0.00 | 0.59 | 0.00 | 0.07 | 0.01 | 0.00 | 0.24 |
| **LB** | 0.00 | 0.00 | 0.00 | 0.00 | 0.00 | 0.01 | 0.07 | 0.00 | 0.12 | 0.12 | 0.24 | 0.00 | 0.11 | 0.00 | 0.12 | 0.19 |
| **MHB** | 0.00 | 0.00 | 0.00 | 0.01 | 0.00 | 0.00 | 0.00 | 0.02 | 0.02 | 0.00 | 0.04 | 0.00 | 0.80 | 0.00 | 0.00 | 0.11 |
| **TSB** | 0.00 | 0.00 | 0.00 | 0.00 | 0.00 | 0.00 | 0.29 | 0.00 | 0.00 | 0.00 | 0.68 | 0.00 | 0.02 | 0.00 | 0.00 | 0.01 |
| **Sa** | 0.00 | 0.00 | 0.00 | 0.00 | 0.00 | 0.00 | 0.20 | 0.00 | 0.03 | 0.03 | 0.55 | 0.00 | 0.09 | 0.00 | 0.00 | 0.10 |
| **Se** | 0.00 | 0.00 | 0.04 | 0.00 | 0.00 | 0.01 | 0.08 | 0.03 | 0.00 | 0.00 | 0.60 | 0.00 | 0.01 | 0.01 | 0.08 | 0.15 |
| **Total** | 0.00 | 0.00 | 0.01 | 0.00 | 0.00 | 0.00 | 0.18 | 0.01 | 0.02 | 0.02 | 0.52 | 0.00 | 0.08 | 0.00 | 0.02 | 0.12 |

**Table S6** Media composition and characteristics

| | BRAIN HEART INFUSION | LYSOGENY BROTH LENNOX | MUELLER HINTON BROTH | TRYPTIC SOY BROTH |
|---|---|---|---|---|
| **Manufacturer Part Number(s)** | Bacto<br>237500 | Bacto<br>211705, 288620 | Difco<br>275730 | Bacto<br>211825 |
| **Protein source(s)** | Proteose Peptone 10.0 g<br>Calf Brain Infusion 7.7 g<br>Beef Heart Infusion 9.8 g | Tryptone 10.0 g | Acid Digest of Casein 17.5 g<br>Beef Extract Powder 2.0 g | Pancreatic Digest of Casein 17.0 g<br>Papaic Digest of Soybean 3.0 g |
| **Sugar/Starch** | Dextrose 2.0 g | | Starch (amylopectin) 1.5 g | Dextrose 2.5 g |
| **Salts** | NaCl 5.0 g | NaCl 5.0 g | | NaCl 5.0 g |
| **Buffer** | $Na_2HPO_4$ 2.5 g | | | $K_2HPO_4$ 2.5 g |
| **Other** | | Yeast extract 5.0 g | | |
| **Notes** | | • No cholesterol<br>• No saturated fatty acids<br>• Yeast extract total carbohydrates = 816 mg | • Not cation-adjusted ($Ca^{2+}$, $Mg^{2+}$)<br>• Low thymine and thymidine | |

**Table S7** Parameters for HS-SPME volatiles sampling, GC×GC-TOF analysis, and data processing and alignment

| **Autosampler Method** | |
|---|---|
| Instrument description | Gerstel® MPS Pro® |
| Software description | Gerstel® Maestro® (version 1.5.3.2) |
| **Sampling Parameters** | |
| Cooled tray temperature | 4 °C |
| Solid-phase microextraction (SPME) | Manufacturer: Supelco®<br>Fiber type: PDMS/CAR/DVB (2 cm; 50/30 μm) |
| Incubation time | 5 min |
| Agitator parameters, incubation | Temperature: 50 °C<br>On time: 10 s<br>Off time: 1 s<br>Speed: 600 rpm |
| Agitation, sampling | On |
| Vial penetration | 21 mm |
| Extraction time | 10 min |
| Injection penetration | 67 mm |
| Desorption time | 180 s |
| **Inlet (CIS) Parameters** | |
| Initial temperature | 250 °C |
| Equilibrium time | 0.05 min |
| Initial time | 0.10 min |
| Ramp rate | 12 °C·s$^{-1}$ |
| End temperature | 250 °C |
| Hold time | 10.5 min |

| **GC×GC Method** | |
|---|---|
| Instrument description | Agilent® 7890B |
| Column configuration | Column 1: Rxi®-624Sil MS, 60 m × 0.25 mm × 1.4 $\mu$m<br>Column 2: Stabilwax®, 1 m × 0.25 mm × 0.5 $\mu$m |
| Carrier gas | Helium, 2 mL·min$^{-1}$ (constant) |
| Front inlet type | Gerstel® |
| Front inlet mode | Splitless |
| Front inlet septum purge flow | 1 mL·min$^{-1}$ |
| Front inlet septum purge time | 300 s |
| Front inlet purge flow | 50 mL·min$^{-1}$ |
| Front inlet total purge flow | 52 mL·min$^{-1}$ |
| Oven equilibration time | 5 s |
| Primary oven temperature ramp | Initial temperature: 35 °C<br>Initial time: 0.5 min<br>Ramp rate: 5 C·min$^{-1}$<br>Final temperature: 230 °C<br>Hold time: 5 min |

| | |
|---|---|
| Secondary oven temperature offset | +5 °C (relative to primary oven) |
| Modulator temperature offset | +15 °C (relative to secondary oven) |
| Modulation timing | Modulation period: 2.00 s<br>Hot pulse time: 0.50 s<br>Cold pulse time: 0.50 s |
| Transfer line temperature | 250 °C |

## Mass Spectrometry Method

| | |
|---|---|
| Instrument description | LECO® Pegasus® 4D |
| Use GC method total time for MS method total time | Yes |
| Acquisition delay | 180 s |
| Filament active time | 180 s to end of run |
| Start mass/End mass | 35/400 |
| Acquisition rate | 100 spectra·$s^{-1}$ |
| Optimized voltage offset | +50 V |
| Electron energy | -70 eV |
| Ion source temperature | 250 °C |
| Acquisition masses | TIC |

## Data Processing Method

| | |
|---|---|
| Software description | LECO® ChromaTOF® and Statistical Compare (version 4.71.0.0) |
| Baseline tracking/Offset | Entire run/0.5 (through middle of noise) |
| Data points averaged for smoothing | Auto |
| First dimension peak width | 12 slices |
| Mass spectral match required to combine peaks | 600 |
| Second dimension peak width | 0.15 |
| Min. subpeak signal-to-noise (S/N) for retention | 6 |
| Integration approach | Traditional |
| Peak finding | S/N: 50<br>Min. number of apexing masses: 2 |
| Mass to use for area/height calculation | Unique mass |
| Alignment analyte match criteria | Spectral match mass threshold: 10<br>Minimum spectral similarity match: 600<br>Max. number of modulation periods apart: 3<br>Max. retention time difference: 0.2<br>S/N for second peak find: 5 |
| Criteria for inclusion of analytes | Min. number of samples that contain analyte: 1 |
